# From Al-Zahrawi’s *Kitab Al-Tasrif* to modern oncology: enduring principles of surgical cancer management

**DOI:** 10.1097/MS9.0000000000004988

**Published:** 2026-05-07

**Authors:** Tareq M. R. Al-Jaberi

**Affiliations:** Faculty of Medicine, Jordan University of Science and Technology and King Abdulla University Hospital, Irbid, Jordan

**Keywords:** Albucasis, Al-Zahrawi, cancer surgery, Kitab al-Tasrif, medieval medicine, surgical oncology

## Abstract

**Introduction:**

Cancer has been recognized as a disease since antiquity, with references documented across multiple civilizations. While the most effective advances in diagnosis and treatment have emerged in recent decades, fundamental principles – particularly the role of surgical excision – were already described and practiced by earlier physicians. Among the most prominent was Abū al-Qāsim Al-Zahrawi (Albucasis, 936–1013 CE), whose encyclopedic treatise *Kitab al-Tasrif* outlined surgical approaches to cancer that reflect enduring principles in oncologic care. This review analyzes Al-Zahrawi’s descriptions of cancer management in *Kitab al-Tasrif* and evaluates their relevance to modern principles of surgical oncology.

**Methods:**

A historical-comparative review of Al-Zahrawi’s descriptions of cancer surgery was undertaken, focusing on his rationale, operative techniques, and cautions. These were compared with contemporary surgical oncology principles to identify continuities and distinctions. Al-Zahrawi advocated complete surgical excision with surrounding healthy tissue, acknowledged the risk of recurrence, emphasized the importance of early diagnosis, and recommended caution in advanced or deeply hidden disease. These insights parallel enduring principles of surgical oncology. Despite the limitations of medieval resources, his writings demonstrate a sophisticated anticipation of doctrines that underpin modern cancer surgery.

**Conclusions:**

Al-Zahrawi’s *Kitab al-Tasrif* illustrates how medieval physicians developed an advanced understanding of cancer surgery. His teachings highlight both the continuity of surgical principles across centuries and the enduring legacy of medieval medical thoughts in shaping modern oncologic practice.

## Introduction

Cancer as a disease has been known to most of the civilizations since antiquity; however, most of the effective advancements in its diagnosis and management have been achieved in the last few decades. Nevertheless, some of the basic principles in its management including surgery have been recognized and practiced by our predecessor physicians.

In this article, we reviewed the chapters on cancer as published in the 10th century medical encyclopedia of the famous Arab Andalusian surgeon Abu Al-Qasim Khalaf ben Abbas Al-Zahrawi Al-Ansari (936–1013), originally from a Medinian tribe of the Arabian Peninsula, and popularly known as Al-Zahrawi, and Latinized as Albucasis or Abulcasis. He lived in Cordoba, the capital of Muslim Spain (Al-Andalus), where he studied, taught, and practiced medicine and surgery[1].

HIGHLIGHTS

- A historical-comparative review.
- It compares Al-Zahrawi’s principles of cancer surgery with contemporary surgical oncology principles.
- These insights proved to be in parallel with enduring principles of surgical oncology.
- This study demonstrates an advanced understanding of cancer surgery by medieval physicians.
- It also demonstrates the enduring legacy of medieval medical thoughts in shaping modern oncologic practice.

**Figure 1.**
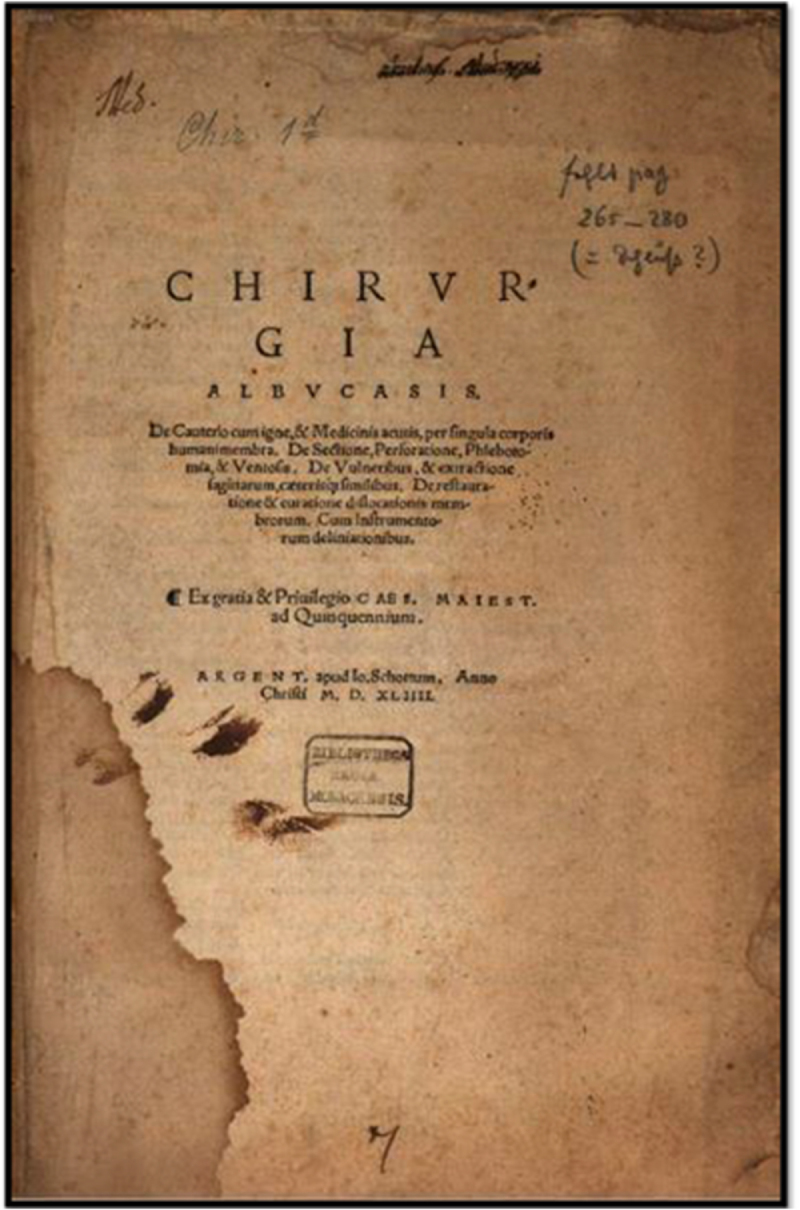
Title page of one of the Latinized translations of the 30th treatise of *Kitab al-Tasrif* printed in Venice in 1544.

**Figure 2.**
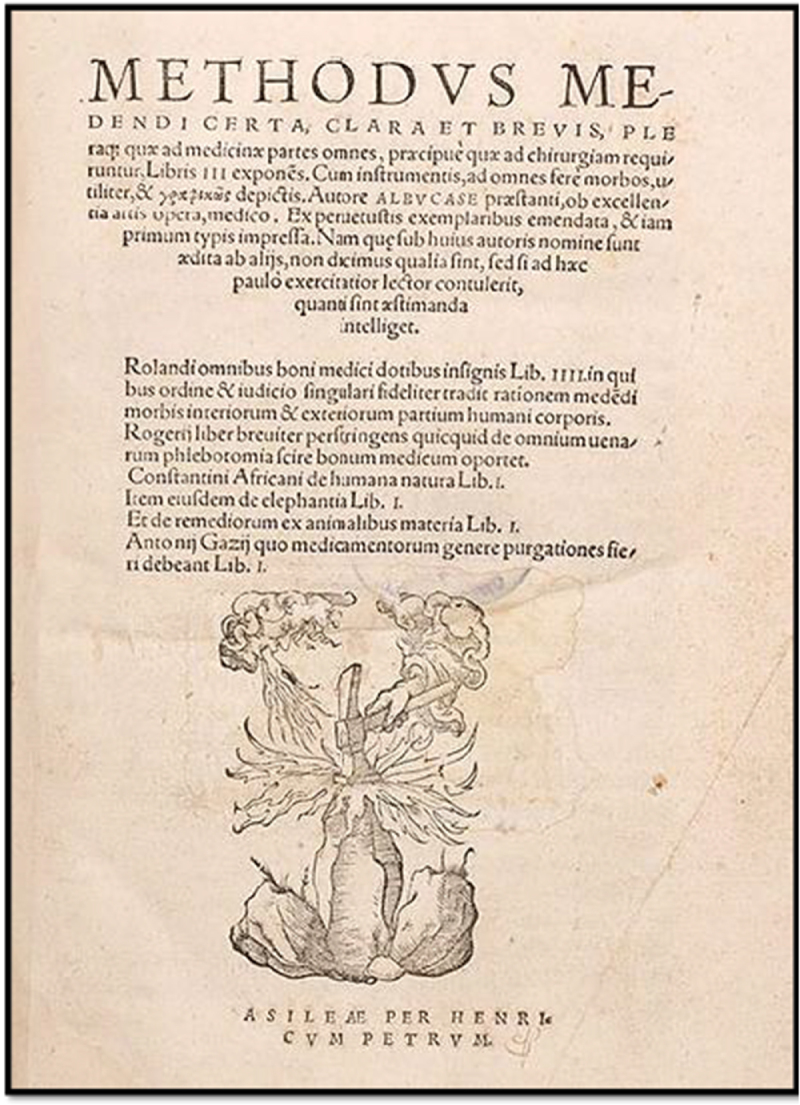
Title page of *Methodus medendi certa, clara, et brevis*, Basel: Henricus Petrus, 1541.

Al-Zahrawi’s principal work, *Kitab al-Tasrif*, a 30-volume encyclopedia of medical practice was completed around the year 1000. It encompasses the Greco-Roman medical legacy and the cumulative experience of centuries of Arab-Islamic medicine. It was disseminated across medieval Europe after the translation of its surgical section, known as “Liber Chirurgiae” into Latin by the renowned 12th-century translator Gerard of Cremona, which introduced European physicians to the legacy of Al-Zahrawi (Figs 1 and 2)[1].

The manuscript of this translation represents the oldest medical treatise written in England and dates back to the year 1250[2]. As interesting, it represents the oldest illustrated printed book in Sibbald Library in London, printed in the last year of the 15th century[3].

His pioneering contributions to surgical procedures and instrumentation had a profound impact not only on medieval medicine but also on modern surgical practice. Of the 200 surgical instruments he described, many reflect principles still in use today. The translated surgical treatise became a standard textbook in European medical schools, most notably at Salerno, Montpellier, and later Bologna, serving as the foundational reference for surgical education for over five centuries^[^2,4–6^]^.

This article adheres to the TITAN 2025 guidelines for the transparent reporting of AI-assisted content[7]. Artificial intelligence was used for language drafting and figure quality improvement.

## Materials and methods

The 30th section of the Encyclopedia was devoted to surgery and composed of three “books” or divisions; each divided into chapters. The manuscript used for our review was that of the year 1541 titled *Methodus medendi certa, clara, et brevis* published in Basel by Henricus Petrus. Ownership marks at the head of the page read *ex Libris Claudii Chucelot Doctoris Medici.* This manuscript is available free online through the Congress library^8^.

In its second book, Chapter 53 was devoted to cancer surgical management. Cancer and its management was also discussed in 10 other sections of the first and second books as part of the specific organ disease management, including the nose, tongue, lips, uvula, skin, and genital organs. We have chosen Chapter 25 dealing with lesions in the nose as a representative of these specific organ treatments. The Latin text of these chapters and their English translations were analyzed. The textbook *Albucasis on Surgery and Instruments: A Definitive Edition of the Arabic Text with English Translation and Commentary* by “Spink and Lewis” was used as the reference for English translation[1].

**Figure 3.**
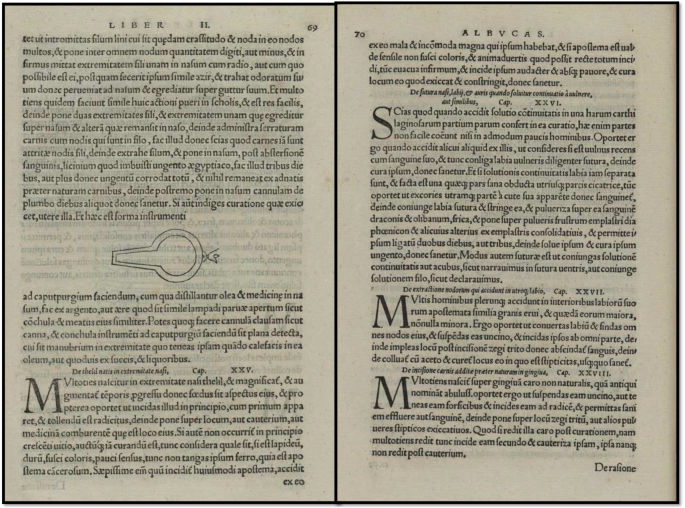
Chapter XXV (23) – On Cancer. From the Latin edition of the surgical work of Albucasis (Abulcasis), *Methodus medendi certa, clara, et brevis* published in Basel by Henricus Petrus in the year 1541, page 264.

Latin text; cap. xxv. de natis in extremitate nasi (Fig. 3).

“Multoties in acutis extremitatibus nasi, & magnificé, & augmentata epotis perigris excrescentiae gignuntur, quae faciei speciem eius, & ipsius oris, deformant. Ideoque oportet, ut cum primum apparuerit, excidantur, & penitus eradantur, donec penitus locus eius excoletur, aut igni, aut medicinis comburentibus, quæ est illoco eius. Si autem non occurrit in principio, sed relinquatur, atque cum creverit consideretur, si durum sit, scilicet coloris pallidi sensusque exiguus, non tangas ipsum ferro, quia est cancerosum. Saepissime enim qui incidit huiusmodi apostema, accidit ut inde major dolor, & in fine nullum adjumentum accidat ei, cui inciditur. Sin vero non fuerit durum, sed mollies, neque coloris pallidi, & viperish ipsum posse ex toto excidi, priusquam excidatur, purges ipsum a males humorists, deinde excide, & postea locus eius curetur medicinis astringentibus, donec convalescat.”

English translation; Chapter 25 on warts growing on the end of the nose (page 264)^[^1^]^.

“There frequently spring from the end of the nose warts that grow and increase daily till they disfigure the man. So, you should cut them out when they first appear; totally eradicate them and apply to the place cauterization, either actual or by caustic. But if excision of them has been overlooked till they have grown big, then examine, and if the growth is stone-like, hard, and pale in color, and with little sensation, do not interfere with it with an instrument, for it is a cancerous tumor. For I have often seen people cut these tumors, and great affliction resulted to the sufferer. But if the tumor be soft to the touch, not pale colored, and you see it can be wholly removed, then purge the patient, and cut it off him fearlessly, and treat the place with astringents and styptics till healed.”

**Figure 4.**
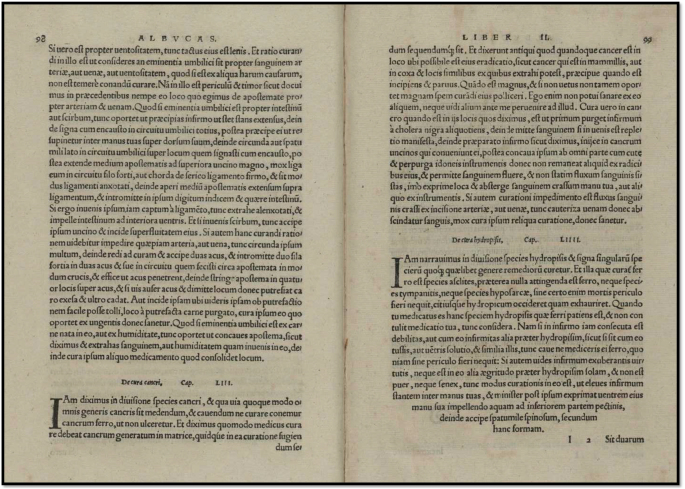
Chapter LIII (53) – On Cancer. From the Latin edition of the surgical work of Albucasis (Abulcasis), *Methodus medendi certa, clara, et brevis* published in Basel by Henricus Petrus in the year 1541, page 380.

Latin text; cap. liii. de cancro et eius curatione (Fig. 4).

“Locuti sumus in loco suo de speciebus cancri, et quomodo eorum curatio tentanda sit: cum admonitione, ne ferro curentur, ne fiant ulcera. Et diximus etiam de cancro uteri, et monuimus ne curetur.

Dixerunt antiqui: Quando cancer est in loco ubi potest radicitus evelli, ut in mammis, vel in coxis, et similibus partibus, ubi totum evelli potest, et maxime si in principio fuerit et parvus, tunc tentanda est curatio ferro. Si vero vetus fuerit et magnus, dimittatur. Ego vero numquam curavi aliquem eorum, nec vidi ante me aliquem curasse.

Et modus curandi, si sit in curabilibus, ut diximus, est ut purgetur prius aegrotus a cholera nigra pluries, deinde si venae plenae fuerint, minuantur. Postea ponatur aegrotus in loco apto operationi. Deinde imponantur fibulae seu unci ad cancrum. Postea faciatur incisio circularis, ita ut comprehendatur cutis accurate, donec radix eius minime remaneat. Et permittatur sanguis fluere, nec cito cohibeatur, sed comprimatur locus, et exprimatur sanguis crassus manu, vel aliquo instrumento. Si autem inciderit vena, vel arteria, et fluxerit sanguis nimis, comburatur vena, vel arteria, donec sistat fluxus. Postea curetur locus more consueto usque ad sanitatem.”

English translation; Chapter 53 on cancer (page 380)[1].

“We have spoken, in the relevant section, of the kinds of cancer and of the way in which medical treatment of them is to be attempted; with a warning against treatment by the knife lest they ulcerate. We also mentioned the cancer arising in the uterus and gave a warning against its treatment.

The Ancients said that when a cancer is in a site where total eradication is possible, such as a cancer of the breast or of the thigh, and in similar parts where complete removal is possible, and especially when in the early stage and small, (then surgery was to be tried). But when it is of long standing and large you should leave it alone. For I myself have never been able to cure any such, nor have I seen anyone else succeed before me. The procedure in a case amenable to treatment, as we have said, is first for the patient to be purged several times from black bile; then bleed him if his veins seem full. Then put the patient in the most convenient position for operating. Then attach to the tumor hooks suited to it; then make a circular incision all round to include the skin with the utmost thoroughness so that not the least root of it remains; let the blood flow and do not stanch it quickly; but put pressure upon the place and squeeze out all the thick blood, either by hand or with any instrument you can devise. If in operating you get a very severe hemorrhage from cutting an artery or vein, cauterize the vessel until the bleeding stops; then dress in the usual way until healed”.

## Discussion

This text clearly demonstrates some of the principles known today about cancer management:

1. The proper diagnosis of cancer before embarking on treatment[9] “if the growth is stone-like, hard, and pale in color, and with little sensation, do not interfere with it with an instrument, for it is a cancerous tumor. For I have often seen people cut these tumors, and great affliction resulted to the sufferer.”
2. Cure can only be achieved when cancer is in an early stage[10], “especially when in the early stage and small, then surgery was to be tried,” this was the experience of Al- Zahrawi and his predecessors; a conclusion corresponding to (stage-dependent outcome) in modern medicine.
3. Surgery is the only curative treatment[11] “cancer must be treated with the knife”; a fact used to be true till the 20th century and remains partially true till now.
4. However, surgery can only be done for accessible areas[11] “in the breasts, or in the hip, or in similar places from which it can be removed.” The site of the tumor and its relations to surroundings remain a major factor in effective surgical treatment.
5. Surgery should be avoided when calculations are not in its favor[12] “if it is large, and cannot be operated upon safely, it is generally extremely dangerous,” a valuable advice of today’s medical practice.
6. Surgery should be radical[13] “where complete removal is possible,” a major principle in today’s practice.
7. Excision should reach healthy tissues[13], “should cut out the cancer on every side until he reaches healthy tissue,” corresponding to the principle of (safe margin) in modern practice.
8. Radicality should include the roots^[^13,14^]^ “utmost thoroughness so that not the least root of it remains”; a primitive statement corresponds to modern (oncological resection).
9. This surgical treatment should be performed after proper preparation[15] “Then, after preparing the patient as we have described,” a valuable modern practice of optimizing the patient’s condition before surgery.
10. Cautery is the major tool to stop bleeding[16] “a very severe hemorrhage from cutting an artery or vein, cauterize the vessel until the bleeding stops”; and cautery remains so in modern practice with modern instruments.

## Evolution of cancer surgery

Early cancer surgery was limited to the excision of superficial and anatomically accessible tumors and was largely palliative in intent. Outcomes were poor due to the absence of anesthesia, lack of antiseptic techniques, and minimal understanding of tumor biology and mechanisms of spread. As a result, surgical intervention was often reserved for symptom relief rather than cure.

A fundamental shift occurred in the mid-19th century with the introduction of general anesthesia in the 1840s and the subsequent adoption of antiseptic principles by Joseph Lister in the 1860s. These developments significantly reduced operative mortality and enabled longer, more complex surgical procedures, allowing surgery to emerge as a viable therapeutic option for cancer rather than a measure of last resort[17].

The late 19th century marked the rise of radical oncologic surgery, driven by the prevailing belief that cancer spread in a contiguous anatomical manner. This concept was most notably exemplified by William Halsted’s radical mastectomy, first reported in 1894, which emphasized en bloc removal of the primary tumor along with surrounding tissues and regional lymph nodes. This approach established the foundational oncologic principle of complete tumor excision with histologically negative margins[18].

During the same period, Theodor Billroth made landmark contributions to cancer surgery. He performed the first successful total laryngectomy for cancer in 1873 and, on 29 January 1881, achieved the first successful resection for gastric antral carcinoma. These procedures demonstrated that internal malignancies could be surgically treated with curative intent, expanding the scope of oncologic surgery beyond superficial disease[19].

Radical surgical principles were further refined in gynecologic oncology. John G. Clark described early radical hysterectomy techniques for cervical cancer in 1895, which were subsequently standardized and popularized by Ernst Wertheim in the early 20th century. Wertheim’s radical hysterectomy became a cornerstone in the curative management of invasive cervical cancer and reinforced the importance of systematic resection of adjacent tissues and lymphatic pathways^[^20,21^]^.

Over time, emphasis shifted from the extent of resection alone to the prognostic significance of surgical margins. The concept of an adequate or “safe” margin remains central to oncologic surgery, although its optimal extent continues to be refined. In rectal cancer, the introduction of total mesorectal excision by Heald and the demonstration by Quirke and colleagues of the prognostic importance of the circumferential resection margin highlighted the critical relationship between margin status and local recurrence^[^22,23^]^. This evolution was facilitated by advances in pathological assessment, including standardized specimen handling, accurate margin orientation, serial sectioning, and improved histopathological techniques, which enabled precise evaluation of resection margins and strengthened the correlation between surgical quality and oncologic outcomes^[^23,24^]^.

Despite advances in surgical technique, perioperative care, and adjuvant therapies, the stage of disease at presentation remains one of the strongest determinants of outcome across most malignancies. This recognition underpins the global emphasis on cancer screening and early detection programs, which aim to identify disease at a stage where complete surgical resection with curative intent is most achievable[25].

## Conclusion

In what was called dark ages, our predecessor physicians have managed cancer in the same basic principles of today, but with primitive resources and tools including absence of anesthesia, lack of antiseptic techniques, and lack of advanced pathological evaluation tools. However, their practice laid doors open for what we have achieved today.

## Data Availability

Data sharing is not applicable to this article as no new datasets were created or analyzed during the current study. All analyzed data are derived from the publicly available sources cited in the reference list.

## Research registration unique identifying number (UIN)

Not applicable (historical review).
